# Impact of platelet-derived mitochondria transfer in the metabolic profiling and progression of metastatic MDA-MB-231 human triple-negative breast cancer cells

**DOI:** 10.3389/fcell.2023.1324158

**Published:** 2024-01-12

**Authors:** Lucas Cereceda, J. Cesar Cardenas, Maroun Khoury, Eduardo Silva-Pavez, Yessia Hidalgo

**Affiliations:** ^1^ IMPACT, Center of Interventional Medicine for Precision and Advanced Cellular Therapy, Santiago, Chile; ^2^ Laboratory of Nano-Regenerative Medicine, Faculty of Medicine, Universidad de los Andes, Santiago, Chile; ^3^ Center for Integrative Biology, Faculty of Sciences, Universidad Mayor, Santiago, Chile; ^4^ Geroscience Center for Brain Health and Metabolism, Santiago, Chile; ^5^ Buck Institute for Research on Aging, Novato, CA, United States; ^6^ Department of Chemistry and Biochemistry, University of California, Santa Barbara, Santa Barbara, CA, United States; ^7^ Cells for Cells and Consorcio Regenero, Chilean Consortium for Regenerative Medicine, Santiago, Chile; ^8^ Facultad de Odontología y Ciencias de la Rehabilitación, Universidad San Sebastián, Bellavista, Santiago, Chile

**Keywords:** platelets, platelet-derived mitochondria, cancer, mitochondria transfer, metabolic adaptability

## Abstract

**Introduction:** An active role of platelets in the progression of triple-negative breast cancer (TNBC) cells has been described. Even the role of platelet-derived extracellular vesicles on the migration of MDA-MB-231 cells has been reported. Interestingly, upon activation, platelets release functional mitochondria into the extracellular environment. However, the impact of these platelet-derived mitochondria on the metabolic properties of MDA-MB-231 cells remains unclear.

**Methods:** MDA-MB-231 and MDA-MB-231-Rho-0 cells were co-cultured with platelets, which were isolated from donor blood. Mitochondrial transfer was assessed through confocal microscopy and flow cytometry, while metabolic analyses were conducted using a Seahorse XF HS Mini Analyzer. The mito-chondrial DNA (mtDNA) copy number was determined via quantitative PCR (qPCR) following platelet co-culture. Finally, cell proliferation and colony formation assay were performed using crystal violet staining.

**Results and Discussion:** We have shown that platelet-derived mitochondria are internalized by MDA-MB-231 cells in co-culture with platelets, increasing ATP production, oxygen (O_2_) consumption rate (OCR), cell proliferation, and metabolic adaptability. Additionally, we observed that MDA-MB-231 cells depleted from mtDNA restore cell proliferation in uridine/pyruvate-free cell culture medium and mitochondrial O_2_ consumption after co-culture with platelets, indicating a reconstitution of mtDNA facilitated by platelet-derived mitochondria. In conclusion, our study provides new insights into the role of platelet-derived mitochondria in the metabolic adaptability and progression of metastatic MDA-MB-231 TNBC cells.

## Introduction

Breast cancer is the most prevalent type among women, leading to an estimated 685,000 deaths in 2020. This number is projected to rise to 7 million by 2040 (Vismara et al., 2022b). Among the various subtypes of breast cancer, triple-negative breast cancer (TNBC) is particularly aggressive and is known for its potential to metastasize (Grasset et al., 2022). TNBC is characterized by the absence of estrogen, progesterone, and human epidermal growth factor receptor 2 (HER2) receptors (Garrido-Castro et al., 2019). This lack of receptors contributes to TNBC’s reduced susceptibility to hormonal and anti-HER2 therapies, posing significant treatment challenges (Garrido-Castro et al., 2019).

Platelets, which are small (2–4 µm) anucleate cellular fragments released by megakaryocytes present in bone marrow, play a significant role in the progression and aggressiveness of cancer (Vismara et al., 2021; Yu et al., 2021; Chen et al., 2023). When activated, they release platelet-derived extracellular vesicles (PEVs), which participate in both physiological and pathological contexts, including cancer (Dovizio et al., 2020; Żmigrodzka et al., 2020). Indeed, PEVs are essential to the complex interactions between platelets and cancer cells, especially breast cancer cells (Vismara et al., 2021). These cancer cells can stimulate platelet activation, subsequently triggering the release of PEVs into the extracellular environment (Gomes et al., 2017; Zarà et al., 2018).

Interestingly, platelets serve as the major reservoir for mitochondria within the circulatory system. Upon activation, platelets release respiratory-competent mitochondria enclosed within vesicles and as free organelles (Boudreau et al., 2014). These organelles, which are essential for ATP production through aerobic respiration and the regulation of cellular metabolism, also play a role in metastasis (Scheid et al., 2021). Despite their significance, the implications of mitochondrial release from activated platelets into TNBC cells, specifically MDA-MB-231 cells, remain unclear.

In this study, we discovered that platelet-derived mitochondria can be internalized by MDA-MB-231 cells in co-culture, increasing ATP production, oxygen (O_2_) consumption rate (OCR), cell proliferation, and metabolic adaptability. Interestingly, we observed that MDA-MB-231 cells depleted of mitochondrial DNA (mtDNA) can restore cell proliferation in uridine/pyruvate-free cell culture medium and regain mitochondrial O_2_ consumption after co-culturing with platelets. This suggests that platelet-derived mitochondria facilitate the replenishment of mtDNA. This discovery provides new insights into the impact of mitochondria derived by platelets on the metabolic adaptability and progression of MDA-MB-231 human metastatic TNBC cells.

## Materials and methods

### Cell culture

MDA-MB-231 cells were cultured using DMEM-HG (Sartorius, Cat. #01-055-1A) supplemented with Penicillin/Streptomycin (P/S) (Corning, Cat. #30-002-CI) 1X, 4 mM glutamine (Corning, Cat. #25-005-CI) and 10% fetal bovine serum (FBS) (Gibco^™^, Cat. #10437-028). MDA-MB-231-Rho-0 (Rho-0) cells were generated as previously described (Rabas et al., 2021) and maintained in DMEM-HG supplemented with 50 μg/mL uridine (Sigma Aldrich, Cat. #U3003), 1 mM pyruvate (Sartorius, Cat. #03-042-1B), P/S-1X, 4 mM glutamine, and 10% FBS. Breast cancer cells were cultured at 37°C with 5% CO_2_ in a humidified incubator.

### Co-culture of platelets with MDA-MB-231 cells

For co-culture experiments, MDA-MB-231 and Rho-0 were seeded in cell culture medium with 10% FBS at approximately 8 × 10^3^ cells/cm^2^ the day before platelets were added. The next day, platelets were added at a final concentration of 1.5 × 10^5^ platelets/µL. In the case of MDA-MB-231, the culture medium was replaced by fresh culture medium without FBS before adding platelets. In the case of Rho-0 cells, the cell culture medium was replaced with the same fresh culture medium before the addition of platelets. Control MDA-MB-231 cells were cultured with the same buffer that contained platelets. MDA-MB-231 cells and platelets were incubated for 24 h. After co-culture, MDA-MB-231 cells were washed twice with PBS-1X to remove platelets. Washed MDA-MB-231 cells were split using TrypLE^™^ Express (Gibco^™^) and manually counted with trypan blue. MDA-MB-231 cells were resuspended in cell culture medium with 10% FBS in all the cases and used for subsequent experiments.

### Platelet isolation

Blood was recollected from the cubital vein of healthy donors using a 21-gauge needle (BD Vacutainer^®^, Cat. #367287) and blood recollection tubes with acid-citrate-dextrose solution A (ACD-A) as anticoagulant (BD Vacutainer^®^, Cat. #364606). The tourniquet was applied only to detect the vein and recollect the first 2.5 mL of blood, which were discarded. Blood was centrifuged at 150 g for 20 min to obtain platelet-rich plasma (PRP). PRP was gently taken to a new tube and was added 1 µM prostaglandin E1 (PGE1) (Focus Biomolecules, Cat. #10-4455). PRP was centrifuged at 150 g for 10 min to remove red blood cells and leukocytes. The supernatant was transferred to a new tube. Platelets were pelleted by centrifugation at 1,500 g for 15 min. All centrifugation steps were done at room temperature (RT) without acceleration and break in a swinging-bucket centrifuge. Platelet pellet was washed twice with platelet wash buffer (PWB) (10 mM sodium citrate, 150 mM NaCl, 1 mM EDTA, 1% (w/v) glucose, pH 7.4) and gently resuspended in tyrode’s buffer (TB) (134 mM NaCl, 12 mM NaHCO_3_, 2.9 mM KCl, 0.34 mM Na_2_HPO_4_, 1 mM MgCl_2_, 10 mM HEPES, 5 mM glucose, pH 7.4) without calcium. Platelets were left at 37°C and counted by flow cytometry using CountBright^™^ Plus Absolute Counting Beads (Cat. #C36995). Before adding platelets to MDA-MB-231 cells, platelet-poor plasma (PPP) was added to platelet preparation to achieve a final concentration of at least 0.05%–0.1% (v/v) in the well.

### Platelet activation

Platelets post isolation or after co-culture were fixed with PFA 2% for 15 min at RT. Fixed platelets were resuspended in PBS-1X plus 2% FBS and stained for 15 min at 4°C with CD42a-FITC (BD Pharmigen^™^, Cat. #558818) (1:200) and CD62p-APC (BD Pharmigen^™^, Cat. #550888) (1:200). Platelets were washed with PBS-1X plus 2% FBS and acquired using a BD FACSCanto^™^ II flow cytometer.

### Mitochondrial transfer assessment

Mitochondrial transfer was assessed by flow cytometry and confocal microscopy. For flow cytometry assessment, MDA-MB-231 cells were previously stained with 5 µM CellTrace^™^ Violet (CTV) (Cat. #C34557) according to the manufacturer’s instructions. For confocal microscopy assessment, MDA-MB-231 cells were previously stained with 150 nM MitoTracker^™^ Deep Red (MTDR) (Cat. #M22426) for 25 min at 37°C, washed and seeded over coverslips. In both cases, platelets, at a concentration of 5 × 10^8^ platelets/mL, were stained with 100 nM MitoTracker^™^ Green (MTG) (Cat. #M7514) for 25 min at 37°C, washed with TB plus 1% BSA and 0.5 µM PGE1 and added to MDA-MB-231 cells. The co-culture system was done as described previously. After 24 h incubation, cells were analyzed using a BD FACSCanto^™^ II flow cytometer or a Leica SP8 AT CIAN (Leica Microsystems) confocal microscopy. MTG does not depend on the mitochondrial membrane potential (ΔΨm), unlike MTDR which does rely on this potential (Agnello et al., 2008; Cottet-Rousselle et al., 2011; Xiao et al., 2016).

### Seahorse XF HS mini analyzer

Multiparameter metabolic analysis of MDA-MB-231 cells was performed in an extracellular flux analyzer (Agilent, United States of America). MDA-MB-231 cells were seeded on XF HS Mini 8-well plates and kept overnight at 37°C in 5% CO_2_ with DMEM-HG. After 48 h, the cell culture medium was replaced with Agilent Seahorse XF DMEM medium pH 7.4 (with 5 mM HEPES) with 10 mM glucose 1 h before the assay. Mitochondrial function (Mito stress test) was evaluated using 2 µM oligomycin (Oligo), 200 nM FCCP, 1 µM rotenone (Rot), and 1 µM antimycin-A (AA).

### ATP levels analysis

ATP analysis was done using the kit CellTiter Glo^®^ Luminescent Cell Viability (Promega, Cat. #G7571). After platelet co-culture, 1 × 10^4^ MDA-MB-231 cells or 5 × 10^3^ sorted MDA-MB-231 cells were added to a 96-well white microplate. Cells were allowed to rest at RT for 30 min. Then, 100 µL of the kit reagent was added, and the plate was incubated for 2 min at 140rpm in an orbital shaker. ATP analysis was done by measuring luminescence in a Biotek^®^ FLx800 luminometer. In the case of platelet-rescued Rho-1, Rho-2, and Rho-3 cells, 5 × 10^3^ cells were seeded in 96 well-white microplates and incubated for 48 h. In parallel, a mirror plate was seeded for protein quantification and normalization. After 48 h, the culture medium was removed, and new medium with 5 µM Oligo (Tocris, Cat. #4110) was added. Cells were incubated for 20 h. After incubation, ATP analysis was conducted as described previously. For protein quantification and normalization, cells were lysed with RIPA-1X (ThermoFisher^™^, Cat. #899000) with protease inhibitor (Sigma Aldrich, Cat. #5056489001), and protein quantification was done using the Pierce^™^ BCA Protein Assay kit (ThermoFisher^™^, Cat. #23225). Data was expressed as ATP luminescence normalized by the number of cells or µg of the total protein.

### Mitochondrial DNA (mtDNA) copy number

The total DNA of MDA-MB-231 cells was manually isolated according to (Laird et al., 1991) with some modifications. Briefly, cells were lysed with lysis buffer (100 mM TRIS-HCl pH 8.5, 5 mM EDTA, 0.2% SDS, 200 mM NaCl, 50 μg/mL proteinase K) at 37°C for several hours. Next, one volume of isopropanol was added to the lysate, and the sample was centrifuged at 16000 g for 20 min at 4°C. The supernatant was discarded, and DNA was resuspended in TE buffer (10 mM TRIS-HCl, 0.1 mM EDTA, pH 8). Relative mitochondrial DNA (mtDNA) levels were determined using quantitative PCR (qPCR) by simultaneous amplification of mtDNA and nuclear DNA (ncDNA). The forward and reverse primers for mtDNA, complementary to mitochondrial encoded NADH dehydrogenase 1 (MT-ND1), were 5′-CAT​GGC​CAA​CCT​CCT​ACT​CCT​C-3′ and 5′-TGG​GGC​CTT​TGC​GTA​GTT​GT-3´. The forward and reverse primers for ncDNA, complementary to ribosomal protein lateral stalk subunit P0 (RPLP0), were 5′-CAA​CGG​GTA​CAA​ACG​AGT​CCT​G-3′ and 5′-AAG​CAG​TAA​GGT​AGA​AGG​CCA​CA-3´. The specificity of primers was confirmed by *in silico* analysis using the UCSC genome browser. Each PCR reaction was run in duplicate and contained 6.25 µL 2X Brilliant SYBR^®^ Green qPCR Master Mix (Cat. #600828–51), 0.19 µL Reference Dye ROX (Cat. #600530–53), 0.375 µL primer forward (final concentration 300 nM), 0.375 µL primer reverse (final concentration 300 nM) and variable volume of UltraPure^™^ Distilled H_2_O (Invitrogen^™^, Cat. #10977–015) and DNA. The combined volume of H_2_O and DNA was 5.3 µL, and 10 ng of DNA was used. PCR was done in a Stratagene Mx3000P (Agilent) by using the following protocol: 95°C (10:00 min) + [95°C (0:30 s) + 61°C (0:30 s)] × 40 cycles +95°C (01:00 min). Results were calculated using the 2*2ˆ∆Ct method (Rooney et al., 2015). Products of qPCR were loaded into a 2% agarose gel, and electrophoresis was performed. Gel was visualized using UV light to confirm the expected amplicon.

### Colony formation assay

A colony formation assay was performed on sorted MDA-MB-231 and Rho-0 cells after platelet co-culture. Sorted MDA-MB-231, 1 × 10^3^ cells were seeded in a 6-well plate with glucose-free DMEM-1X (Sigma-Aldrich^®^, Cat. #D5030) supplemented with 10 mM galactose, 4 mM glutamine, and 10% FBS, and grew for 12–13 days. Colonies were visualized by staining with 0.5% crystal violet for 45 min. In the case of Rho-0 cells, 5 × 10^3^ cells after platelet co-culture were seeded in a 6-well plate with medium supplemented with uridine and pyruvate. Cancer cells were maintained for 48 h in cell culture medium with uridine and pyruvate. Then, cell culture medium was replaced with uridine/pyruvate-free DMEM-HG. Cells were cultured for 20 days without uridine/pyruvate, and colony isolation was performed. Colonies were visualized by staining with 0.5% crystal violet for 45 min. Isolated Rho-0 colonies were recovered by platelet co-culture and maintained in uridine/pyruvate-free DMEM-HG.

### Proteomic analysis

Protein data sets related to PEVs-THR, PEVs-BCC, and EVs-MDA were generated by (Vismara et al., 2022a). Mitochondrial proteins in each sample were obtained by comparing every sample with the mitochondrial proteins in the Human MitoCarta 3.0 (Rath et al., 2021). Each sample’s list of identified mitochondrial proteins was compared with the others to identify common and unique ones. Afterward, GO-annotation analysis was performed using STRING V11.5 (Szklarczyk et al., 2019) gene ontology tools. Biological processes with strength >1 and false discovery rate (FDR) < 0.5 were selected.

### Mitochondrial membrane potential (ΔΨm)

The co-culture system was done as described previously, and MDA-MB-231 cells were stained with CTV. Mitochondrial membrane potential was analyzed in MDA-MB-231 cells after platelet co-culture using tetramethyl rhodamine, ethyl ester, and perchlorate (TMRE) (Invitrogen^™^, Cat. #T669), a dye that is dependent on mitochondrial membrane potential. MDA-MB-231 cells were washed with PBS-1X plus 2% FBS, resuspended in PBS-1X plus 2% FBS, and stained with Ghost Dye^™^ Violet 510 (Tonbo^™^, Cat. #13-0870-T100) (1:1,000) for 20 min at 4°C. After that, cells were washed with PBS-1X plus 2% FBS, resuspended in PBS-1X, and stained with 100 nM TMRE for 30 min at 37°C. MDA-MB-231 cells were washed with PBS-1X plus 2% FBS and analyzed using a BD FACSCanto^™^ II flow cytometer.

### Cell proliferation assay

After co-culture, 2.5 × 10^3^ MDA-MB-231 cells in DMEM-HG with 10% FBS were seeded in duplicate in a 96-well flat bottom microplate. At 24, 48, and 72 h, cell proliferation was assessed by 0.5% crystal violet staining for 45 min. Crystal violet was washed, and the plates were allowed to dry out. Next, 200 µL methanol was added to the plate, and absorbance was recorded at 570 nm using an Infinite^®^ 200 NanoQuant plate reader (Tecan^™^).

### Cell sorting

As described previously, platelets were stained with MTG and co-cultured with MDA-MB-231 cells. After co-culture, MDA-MB-231 cells that acquired platelet-derived mitochondria were identified as MTG-positive cells. The MTG-positive cells were separated based on MTG fluorescence intensity using a BD FACSAria^™^ Fusion Cell Sorter. Two subpopulations were recovered: those with high MTG intensity, named MITO-High, and those with low MTG intensity, named MITO-Low. As a control, MDA-MB-231 cells cultured without platelets were subject to the same cell sorting process.

### Data analysis

Flow cytometry data was acquired using the FACS Diva program and analyzed using the FlowJo V10 software. The strategies used to analyze the flow cytometry data associated with each experiment are presented in Supplementary Figure S1. Confocal microscopy images were acquired and analyzed using LAS X software. The area fraction of colonies was measured using ImageJ software. Statistical analysis was performed using GraphPad Prisma 8 version 8.0.2 (GraphPad Software, San Diego, CA, United States of America). For statistical analysis, the t-student statistical test was performed. Results are presented as mean ± SEM. Significance level of statistical tests: *p < 0.05; **p < 0.01; ***p < 0.001 and ns: not significant.

## Results

### Mitochondrial transfer from platelets to MDA-MB-231 breast cancer cells

**FIGURE 1:**
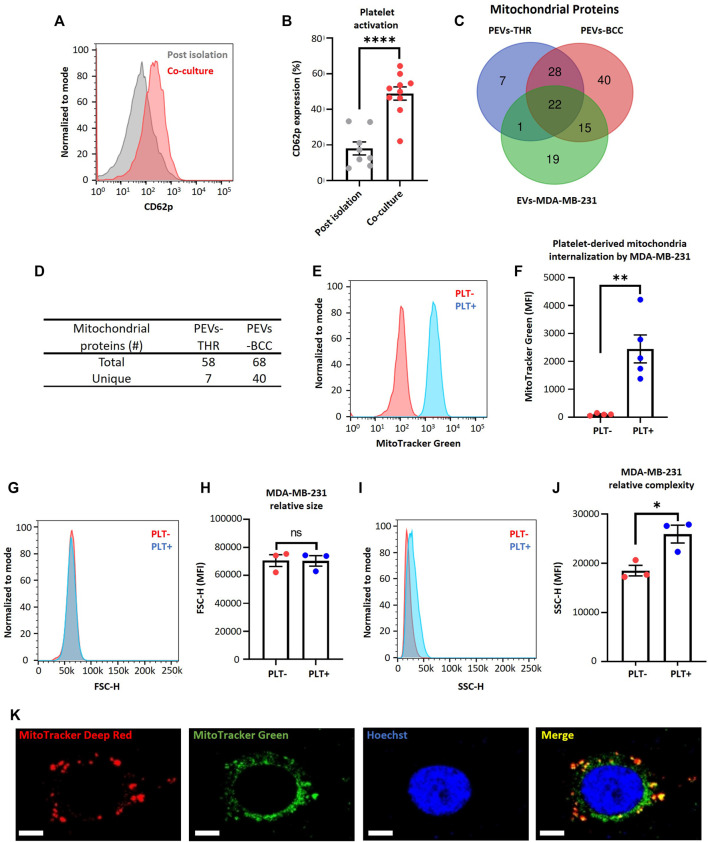
MDA-MB-231 cells acquire platelet-derived mitochondria. **(A)** Flow cytometry histograms of CD62p expression in platelets after isolation or after 24 h co-culture with MDA-MB-231 cells. **(B)** Frequency of platelet activation after isolation or after 24 h co-culture with MDA-MB-231 cells. **(C)** Venn diagram of mitochondrial proteins from three samples: PEVs-THR, PEVs-BCC, and EVs-MDA-MB-231. **(D)** Amount of total and unique mitochondrial proteins for PEVs-THR and PEVs-BCC. Common mitochondrial proteins between PEVs-BCC and EVs-MDA-MB-231 were discarded to avoid possible contamination with proteins from EVs released by MDA-MB-231 cells. **(E)** Flow cytometry histograms of MitoTracker^™^ Green (MTG) signal on MDA-MB-231 cells after 24 h co-culture. MDA-MB-231 cells were cultured with buffer or platelets previously stained with MTG. **(F)** MTG MFI on MDA-MB-231 cells cultured with buffer or MTG platelets. **(G)** Flow cytometry histograms of FSC-H for MDA-MB-231 cells cultured with buffer or platelets for 24 h. **(H)** FSC-H MFI for MDA-MB-231 cells cultured with buffer or platelets for 24 h. **(I)** Flow cytometry histograms of SSC-H for MDA-MB-231 cells cultured with buffer or platelets for 24 h. **(J)** SSC-H MFI for MDA-MB-231 cells cultured with buffer or platelets 24 h. **(K)** Confocal microscopy images of platelet-derived mitochondria internalization by MDA-MB-231 cells. These cells were previously stained with MitoTracker^™^ Deep Red (MTDR) and co-cultured with MTG platelets for 24 h. Scale bar = 10 µm. A t-student statistical test was performed. *p < 0.05; **p < 0.01; ****p < 0.0001; ns = not significant.

The platelet activation by MDA-MB-231 cells has been previously described (Vismara et al., 2022a). Interestingly, the activation of platelets by MDA-MB-231 cells leads to the release of platelet-derived vesicles into the extracellular environment (Zarà et al., 2017). These platelet-derived microparticles play a fundamental role in the complex interaction between the hemostatic system and cancer progression (Vismara et al., 2021). We consistently observed a significant increase in the platelet activation marker (CD62p) on the plasma membrane of platelets, using cytometry flow upon co-culturing with MDA-MB-231 cells compared to the post-isolation platelets (Figures 1A, B).

In addition, retrospective analysis of previously published data (Vismara et al., 2022a), as verified through liquid chromatography-tandem mass spectrometry, identified that the protein constituents of two subpopulations of platelet-derived extracellular vesicles (PEVs) in response to thrombin exposure (PEVs-THR) and during co-culture with MDA-MB-231 cells (PEVs-BCC) were associated with mitochondrial function (Vismara et al., 2022a) (Figures 1C, D). Interestingly, PEVs-BCC exhibited a higher total amount of mitochondrial proteins than PEVs-THR and an increased amount of unique mitochondrial proteins exclusively associated with this condition (Figures 1C, D). These results suggest that platelets likely release mitochondria when activated by MDA-MB-231 cells.

To verify that MDA-MB-231 cells can acquire platelet-derived mitochondria, we stained mitochondria platelets with MitoTracker^™^ Green (MTG) dye, and then these platelets were co-cultured with MDA-MB-231 cells. Notably, the platelet-derived mitochondria internalization in MDA-MB-231 cells was confirmed using flow cytometry (Figures 1E, F). Additionally, we managed to rule out leakage of MTG from platelets to MDA-MB-231 cells (Supplementary Figure S2). These findings are consistent with previous studies associating platelet activation and release of platelet-derived mitochondria (Boudreau et al., 2014). To confirm that stained platelets did not adhere to the cell surface of MDA-MB-231 cells, which could increase their size, we analyzed the size of MDA-MB-231 cells after co-culture with platelets. As shown in (Figures 1G, H), there was no relative change in the size of MDA-MB-231 cells. Nonetheless, it is essential to note that the internal complexity within the MDA-MB-231 cells was considerably increased upon co-culture with platelets (Figures 1I, J). This observation suggests the possible internalization of platelet-derived vesicles and platelet-derived mitochondria within MDA-MB-231 cells (Zoellner et al., 2019).

Furthermore, we employed confocal microscopy to assess the internalization of platelet-derived mitochondria by MDA-MB-231 cells (Figure 1K). We used MTG dye to stain the mitochondria of platelets and MitoTracker^™^ Deep Red (MTDR) to stain the mitochondria of MDA-MB-231 cells, followed by co-culture. Surprisingly, we observed co-localization of platelet-derived mitochondria (green) with mitochondria of MDA-MB-231 cells (red) (Figure 1K). In summary, these findings evidence the transfer of mitochondria *in vitro* from activated platelets to metastatic MDA-MB-231 cells.

### Effect of platelet-derived mitochondria on the mitochondrial function in MDA-MB-231 cells

**FIGURE 2:**
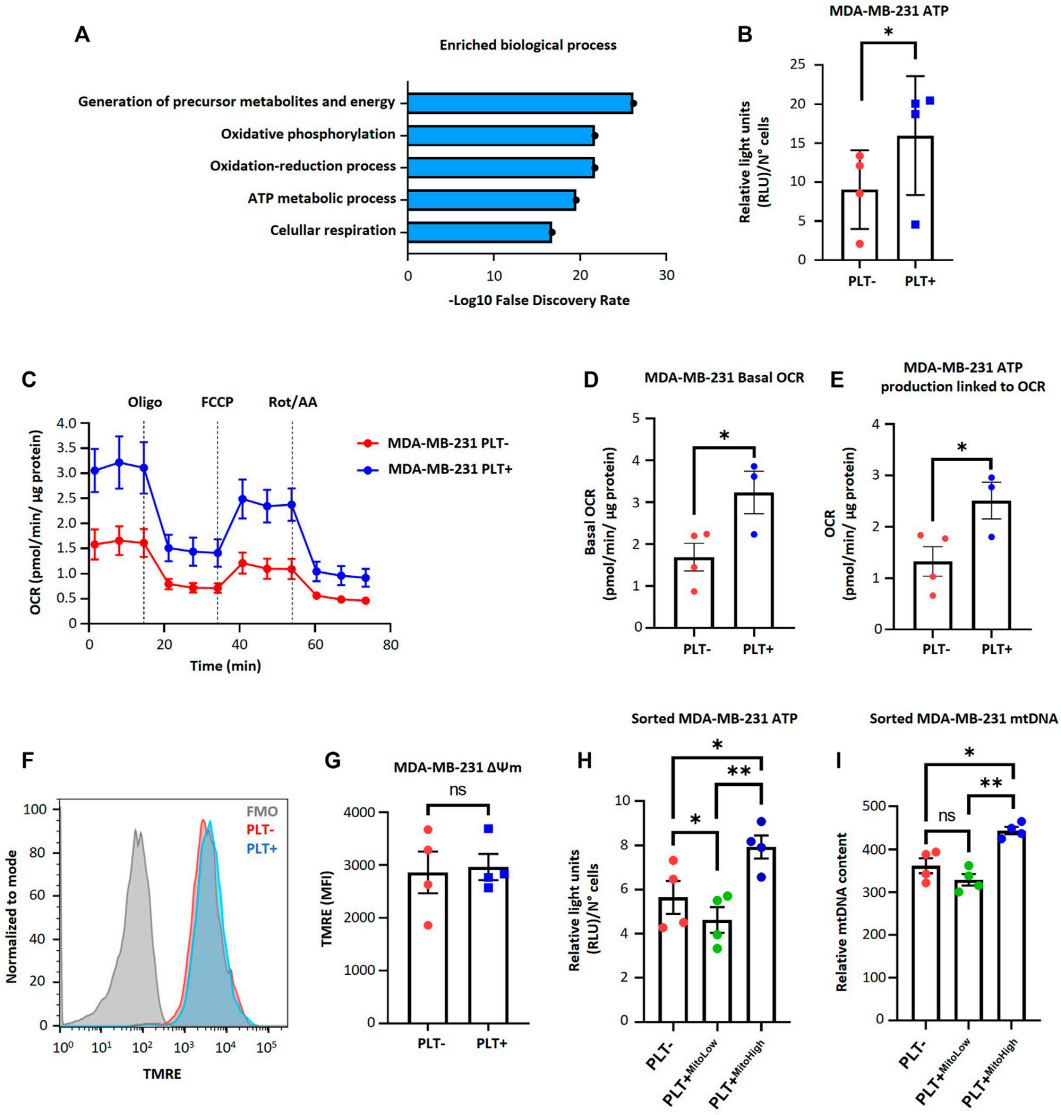
Effect of platelet-derived mitochondria on the bioenergetic properties of MDA-MB-231 cells. **(A)** Gene ontology results of enriched biological process identified for mitochondrial proteins related to PEVs-BCC. GO-annotation analysis was performed using STRING V11.5 gene ontology tools. Biological processes with strength >1 and false discovery rate (FDR) < 0.05 were selected. **(B)** Analysis of ATP content of MDA-MB-231 cells cultured with buffer or platelets for 24 h. Results were expressed as relative light units (RLU) normalized by a total number of cells. **(C)** Seahorse assay results of OCR for MDA-MB-231 cells cultured with buffer or platelets. MDA-MB-231 cells were co-cultured with platelets for 24 h. After that, cells were washed, reseeded, and cultured for 48 h before performing the seahorse assay. Results were normalized to µg of a total protein of each well. **(D)** Basal OCR of MDA-MB-231 cells cultured with buffer or platelets. **(E)** ATP-linked OCR of MDA-MB-231 cells cultured with buffer or platelets. **(F)** Flow cytometry histograms of TMRE signal in MDA-MB-231 cells after 24 h co-culture. MDA-MB-231 cells were cultured with buffer or platelets, and mitochondrial membrane potential (ΔΨm) was analyzed using TMRE dye. **(G)** TMRE MFI in MDA-MB-231 cells cultured with buffer or platelets. **(H)** Analysis of ATP content of sorted MDA-MB-231 cells cultured with buffer or platelets for 24 h. MDA-MB-231 cells were cultured with platelets previously stained with MTG. MDA-MB-231 cells positive for MTG signal were separated according to MTG intensity, retrieving two populations. Those with low MTG intensity are named MITO-Low, and those with high MTG intensity are named MITO-High. Results are expressed as RLU normalized by the total number of cells. **(I)** Analysis of relative mitochondrial DNA (mtDNA) levels of sorted MDA-MB-231 cells. Relative mtDNA levels were determined using quantitative PCR (qPCR) by amplifying mtDNA and nuclear DNA (ncDNA). A t-student statistical test was performed. *p < 0.05; **p < 0.01; ns = not significant.

To understand the metabolic impact of the internalization of platelet-derived mitochondria by MDA-MB-231 cells, we examined the 68 mitochondrial proteins previously identified in the PEVs-BCC condition using gene ontology (GO)-annotation analysis (Figure 2A). Our results indicated that the most significantly enriched processes are inherently associated with mitochondria-associated respiration and energy metabolism (Figure 2A). Next, we evaluated ATP levels in MDA-MB-231 cells after co-culture with platelets. As shown in (Figure 2B), a significant increase in ATP levels was observed in MDA-MB-231 cells after co-culture with platelets. These findings suggest increased energy production within MDA-MB-231 cells after co-culture with platelets. To further investigate the functional impact of mitochondrial transfer, we used a Seahorse XF HS Mini Analyzer to assess mitochondrial bioenergetics of MDA-MB-231 cells during co-culture with platelets. As shown in (Figures 2C–E), there is a significant increase in basal OCR and ATP-linked OCR, indicating a functional effect of the transfer of mitochondria from activated platelets to MDA-MB-231 cells. Next, we evaluated mitochondrial membrane potential (ΔΨm) in MDA-MB-231 cells after co-culture with platelets. As shown in (Figures 2F, G), no significant differences were observed between MDA-MB-231 cells cultured in the presence or absence of platelets.

Additionally, we labeled platelet mitochondria using MTG dye before co-culturing them with MDA-MB-231 cells. Subsequently, we identified MDA-MB-231 cells that internalized platelet-derived mitochondria as MTG-positive cells. These cells were categorized based on MTG fluorescence intensity using cell sorting, resulting in two distinct subpopulations. One subpopulation exhibited high fluorescence intensity of MTG, which we designated MITO-High, while the other subpopulation had low intensity and was designated MITO-Low. In addition, we subjected MDA-MB-231 WT cells cultured without platelets to the same cell sorting procedure for comparison. As shown in (Figure 2H), MITO-High cells had significantly higher ATP content than MITO-Low cells and the MDA-MB-231 cells cultured without platelets. We repeated the abovementioned procedure to confirm whether the increase in ATP production was associated with increased mitochondria within MDA-MB-231 cells. Notably, MITO-High cells possessed more copies of mitochondrial DNA (mtDNA) than MITO-Low and MDA-MB-231 cells cultured without platelets (Figure 2I), suggesting a likely more significant number of mitochondria within MITO-High cells.

### Mitochondrial DNA-deficient MDA-MB-231 cells restore impaired mitochondrial function through acquisition of platelet-derived mitochondria

**FIGURE 3:**
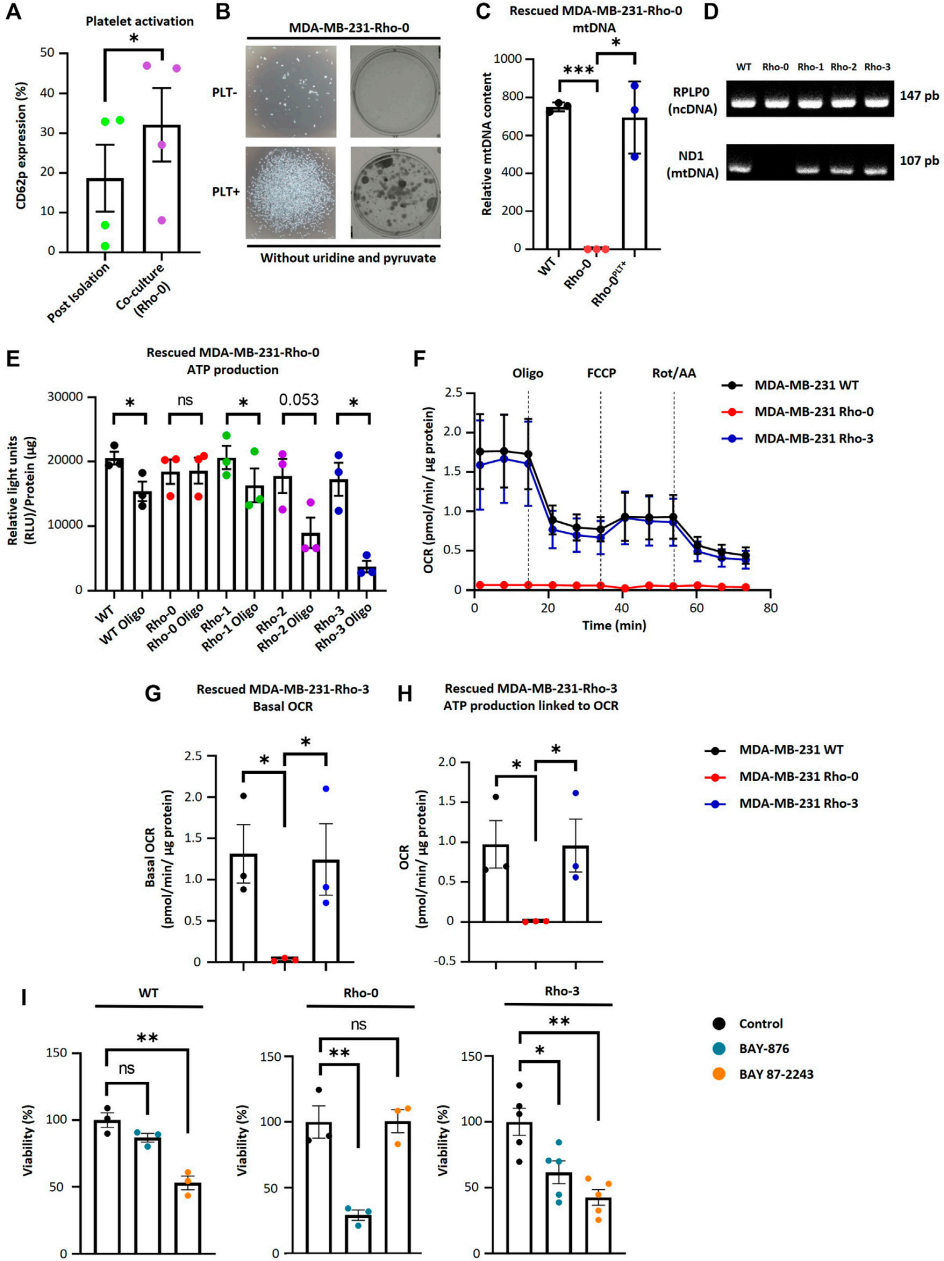
MDA-MB-231-Rho-0 cells restore impaired mitochondrial function by acquiring platelet-derived mitochondria. **(A)** Frequency of platelet activation after isolation or after 24 h co-culture with Rho-0 cells. **(B)** Microscopic (left panel) and macroscopic (right panel) images of colonies formed by platelet-rescued Rho-0 cells in pyruvate/uridine-free cell culture medium. Rho-0 cells were cultured with buffer or platelets for 24 h. After that, cells were washed, reseeded, and cultured for 48 h in medium with pyruvate/uridine. Next, cells were maintained in pyruvate/uridine-free medium to evidence mitochondrial function recovery and colony isolation was performed. Colonies were visualized by 0.5% crystal violet staining. **(C)** Analysis of relative mtDNA levels of platelet-rescued Rho-0 cells by three different donors (Rho-1, Rho-2, and Rho-3). Relative mtDNA levels were determined using quantitative PCR (qPCR) by simultaneous amplification of mtDNA and ncDNA. **(D)** Gel agarose electrophoresis for amplified products of mitochondrial encoded NADH dehydrogenase 1 (MT-ND1) gene and ribosomal protein lateral stalk subunit P0 (RPLP0) gene. Products of qPCR were loaded into a 2% agarose gel, and electrophoresis was performed. Gel was visualized using UV light to confirm the expected amplicon for each primer pair. **(E)** ATP production analysis of platelet-rescued Rho-1, Rho-2, and Rho-3 cells. ATP production was modulated with 5 µM oligomycin (Oligo) for 20 h. Results were expressed as RLU normalized to µg of total protein. **(F)** Seahorse assay results of OCR for platelet-rescued Rho-3 cells. Results were normalized to µg of the protein of each well. **(G)** Basal OCR of platelet-rescued Rho-3 cells. **(H)** ATP production linked to OCR of platelet-rescued Rho-3 cells. **(I)** Cell viability assay of MDA-MB-231 WT, Rho-0, and platelet-rescued Rho-3. Cells were treated with BAY-876 or BAY 87–2243 for 72 h as control cells were treated with vehicle (DMSO). Cell viability was determined by 0.5% crystal violet stained. Results were normalized to control. A t-student statistical test was performed. *p < 0.05; **p < 0.01; ****p < 0.0001; ns = not significant.

To study the functional significance of mitochondrial transfer from activated platelets to MDA-MB-231 cells, we generated MDA-MB-231 cells depleted of mtDNA (Rho-0), as described in Rabas et al. (2021). Rho-0 relies on uridine and pyruvate for their growth due to the absence of a functional respiratory chain (King and Atrardi, 1989; Kukat et al., 2008). The absence of these two metabolic requirements was a selectable marker for reintroducing exogenous mitochondria into Rho-0 cells by co-culture with platelets. We performed co-culture experiments between Rho-0 cells and platelets. As shown in (Figure 3A), we observed a significant increase in platelet activation when co-cultured with Rho-0 cells compared to the post-isolation platelets.

Furthermore, we performed a colony formation assay to confirm whether Rho-0 cells can acquire mitochondria derived from activated platelets. We cultured Rho-0 cells with and without platelets in uridine/pyruvate-free cell culture medium (Figure 3B). Surprisingly, Rho-0 cells cultured with platelets could form colonies in uridine/pyruvate-free cell culture medium. This raised the question of whether these cells successfully repopulated with platelet-derived mitochondria, thus allowing the growth of Rho-0 cells (Figures 3C, D). Indeed, Rho-0 cells cultured with platelets were found to repopulate with platelet-derived mtDNA from the three healthy donors (i.e., platelet-rescued Rho-1, Rho-2, and Rho-3 cells).

To investigate the functional consequences of transferring platelet-derived mitochondria from three donors to Rho-0 cells, specifically on ATP production and OCR. We used oligomycin, a well-known ATP synthase inhibitor, in platelet-rescued Rho-0, Rho-1, Rho-2, and Rho-3 cells. Interestingly, all cell lines showed decreased ATP production after oligomycin (Oligo) treatment, except for Rho-0 cells (Figure 3E). Additionally, we used a Seahorse XF HS Mini Analyzer to assess the mitochondrial bioenergetics of platelet-rescued Rho-3 cells compared to MDA-MB-231 WT and Rho-0 cells. Surprisingly, as shown in (Figures 3F–H), there is a significant increase in basal OCR and ATP-linked OCR, indicating a functional effect of mitochondria transfer from activated platelets in Rho-3 cells compared to Rho-0 cells. Interestingly, in platelet-rescued Rho-3 cells, no differences in basal OCR and ATP-linked OCR were observed with MDA-MB-231 WT cells.

We evaluated the cell viability in MDA-MB-231 WT, Rho-0, and Rho-3 cells following treatment with GLUT-1 inhibitor (BAY-876) and the inhibitor of mitochondrial complex I (BAY 87–2243) (Figure 3I). Notably, using BAY-876 resulted in a significant decrease in the viability of Rho-0 cells, suggesting that these cells rely on glucose metabolism for survival. In contrast, the viability of Rho-0 cells remained stable after treatment with BAY 87–2243, while both MDA-MB-231 WT and platelet-rescued Rho-3 cells showed a marked reduction in cell viability. This decrease in cell viability could be attributed to their oxidative profile, likely resulting from the internalization of platelet-derived mitochondria.

### Platelet-derived mitochondria promote proliferation and increase clonogenic potential by augmenting the metabolic adaptability of MDA-MB-231 cells

**FIGURE 4:**
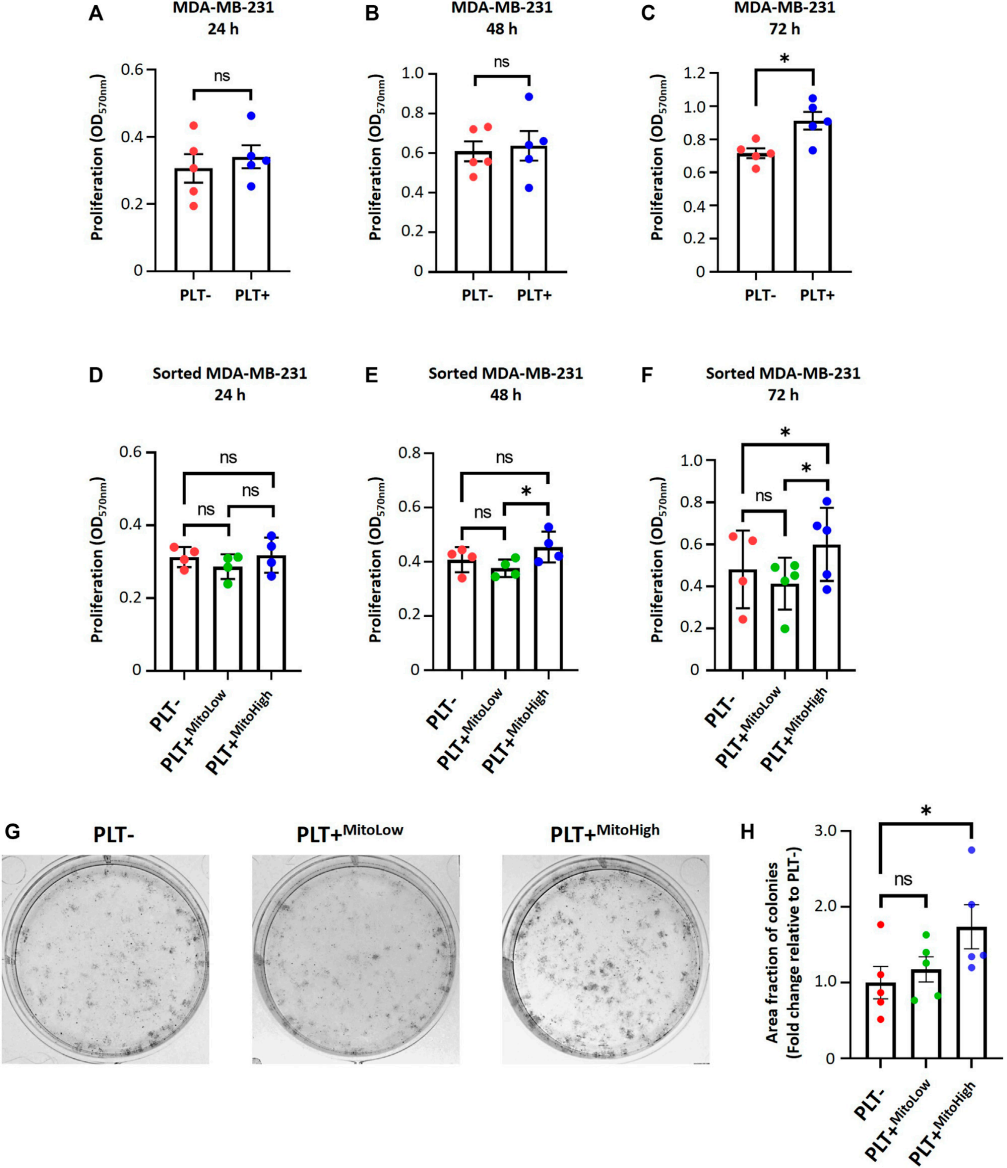
Platelet-derived mitochondria promote proliferation and increase clonogenic potential by augmenting the metabolic adaptability of MDA-MB-231 cells. Proliferation analysis of MDA-MB-231 cells cultured with buffer or platelets for 24 h. MDA-MB-231 cells were co-cultured with platelets. After that, cells were washed, reseeded, and proliferation was evaluated at 24 **(A)**, 48 **(B)**, and 72 h **(C)** by 0.5% crystal violet staining. Cell proliferation assay of sorted MDA-MB-231 cells cultured with buffer or platelets for 24 h. MDA-MB-231 cells were cultured with platelets previously stained with MTG. MDA-MB-231 cells positive for MTG signal were separated according to MTG intensity, retrieving two populations. Those with low MTG intensity are named MITO-Low, and those with high MTG intensity are named MITO-High. Sorted cells were seeded, and proliferation was evaluated at 24 **(D)**, 48 **(E)**, and 72 h **(F)** by 0.5% crystal violet staining. **(G)** Macroscopic images of colonies formed by sorted MDA-MB-231 cells in a glucose-free culture medium with 10 mM galactose and 4 mM glutamine. Sorted cells were cultured in a glucose-free cell culture medium to evidence metabolic adaptability, and colony visualization was performed by 0.5% crystal violet staining. **(H)** Quantification of the area covered by colonies formed in a glucose-free culture medium with 10 mM galactose and 4 mM glutamine. Results were expressed as fold change relative to PLT- (without platelets). A t-student statistical test was performed. *p < 0.05; ns = not significant.

To study the functional significance of internalization of platelet-derived mitochondria on cell proliferation and metabolic adaptation of MDA-MB-231 cells. First, we examined the proliferation of MDA-MB-231 cells in co-culture with platelets. Cell proliferation was evaluated using crystal violet staining at different times following the co-culture (Figures 4A–C). As shown in (Figure 4C), MDA-MB-231 cells cultured with platelets exhibited a significant increase in cell proliferation at 72 h compared to MDA-MB-231 cells not co-cultured with platelets. In the same way as Figure 2H, we assessed the cell proliferation of the MITO-High and the MITO-Low cell subpopulations and MDA-MB-231 cells cultured without platelets (Figures 4D–F). As shown in (Figures 4E, F), the MITO-High subpopulation significantly increased cell proliferation compared to the MITO-Low cells and MDA-MB-231 cells not co-cultured with platelets.

**FIGURE 5:**
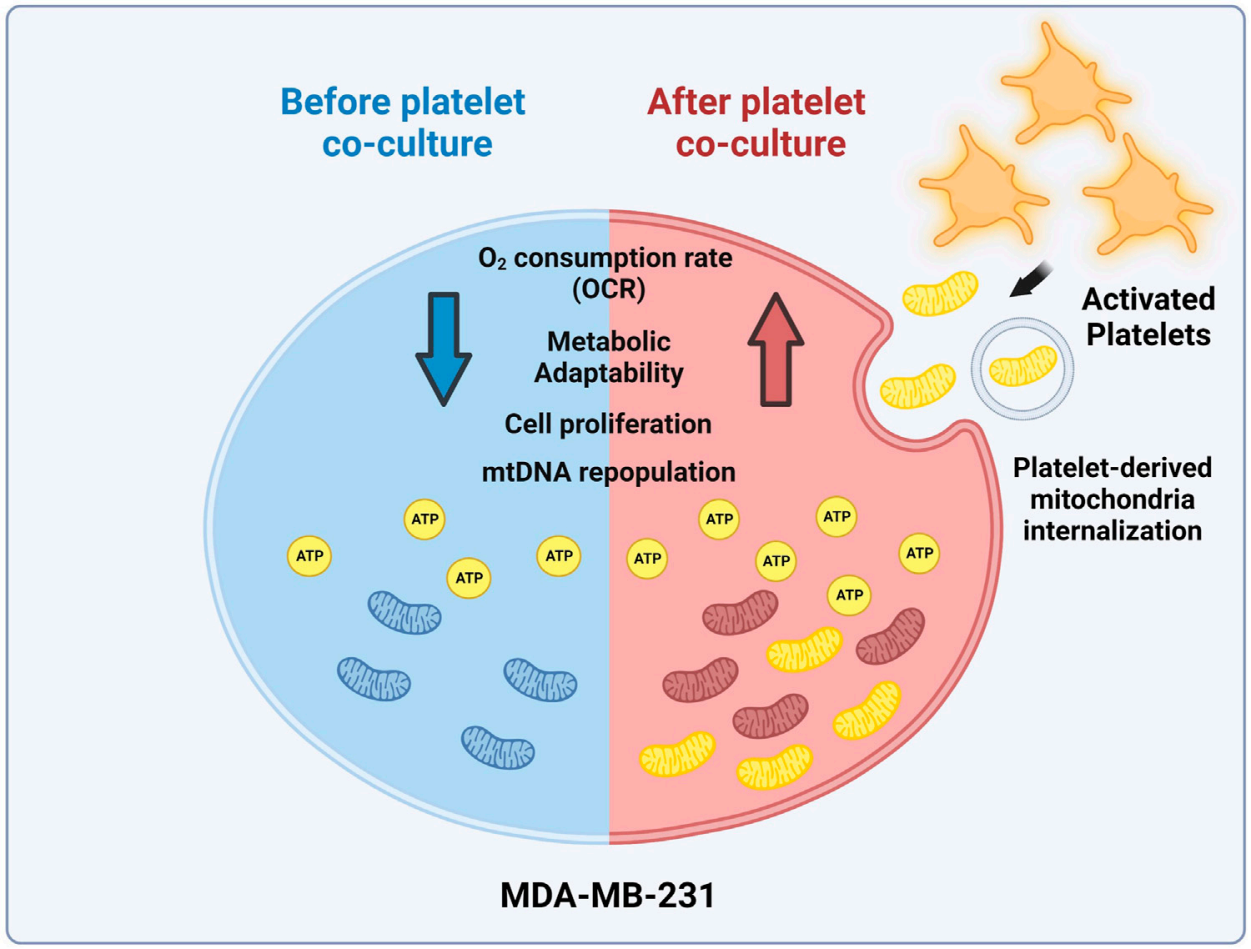
Model of mitochondrial transfer from platelets to MDA-MB-231 cells. Activation of platelets by MDA-MB-231 human triple-negative breast cancer (TNBC) cells triggers the release of platelet-derived mitochondria. These cells then internalize these mitochondria, leading to the reconstitution of mitochondrial DNA (mtDNA), increasing ATP production, oxygen (O_2_) consumption rate (OCR), and cell proliferation. Interestingly, MDA-MB-231 cells with high levels of platelet-derived mitochondria exhibit superior metabolic adaptability. This highlights the significant influence of platelet-derived mitochondria on the progression of MDA-MB-231 TNBC cells (Created with BioRender.com).

In addition, we performed a clonogenic assay using the MITO-High and MITO-Low subpopulations to assess the metabolic adaptability of MDA-MB-231 cells cultured with platelets. For this assay, we used a culture medium without glucose but supplemented with 10 mM galactose and 4 mM glutamine to induce a metabolic shift towards a more oxidative phenotype (Urra et al., 2018). As shown in (Figures 4G, H), we observed that the populations that covered the most area of the plate were MITO-High cells compared to MITO-Low and MDA-MB-231 cultured without platelets. This result confirms an adaptation towards a more oxidative metabolism, possibly due to the more significant number of mitochondria in MITO-high cells. Altogether, our data demonstrate that platelet-derived mitochondria increase cell proliferation and metabolic adaptability of MDA-MB-231 TNBC cells (Figure 5).

## Conclusion

We have demonstrated that platelet-derived mitochondria can be internalized by MDA-MB-231 cells in co-culture, leading to increased ATP production, oxygen (O_2_) consumption rate (OCR), cell proliferation, and metabolic adaptability. Interestingly, we observed that MDA-MB-231 cells depleted from mitochondrial DNA (mtDNA) recover cell proliferation in uridine/pyruvate-free cell culture medium and mitochondrial oxygen (O_2_) consumption after co-culture with platelets, indicating a replenishment of mtDNA facilitated by platelet-derived mitochondria. Altogether, this finding provides new insight into the role of mitochondria derived by platelets in the metabolic adaptability and progression of metastatic MDA-MB-231 human triple-negative breast cancer (TNBC) cells.

## Discussion

Interactions between cancer cells and platelets are well-known phenomena. Upon entering the circulatory system, tumor cells activate and aggregate platelets to evade the immune system and shield themselves from the shear forces of blood flow. This process is crucial for the survival of cancer cells in the circulatory system (Bambace and Holmes, 2011). Recently, it has been described that cancer cells enhance their tumor potential by acquiring exogenous mitochondria (Watson et al., 2023). However, the role of mitochondria-derived from platelets in influencing the metabolic and functional properties of MDA-MB-231 triple-negative breast cancer (TNBC) cells remains unclear.

Initially, we verified through flow cytometry that platelets become activated when co-cultured with MDA-MB-231 cells. However, the specific mechanism of platelet activation employed by MDA-MB-231 cells remained unidentified in our study. It is worth noting that a variety of mechanisms, including the use of pro-coagulant proteins, molecules such as ADP, and the release of inflammatory cytokines and microparticles, have been described for the activation of platelets by tumor cells (Razak et al., 2018). Additionally, previous research has shown that breast cancer cells, specifically MDA-MB-231 cells, can induce platelet activation. This process has been linked to the production of thrombin from plasma and is facilitated by the release of ADP from dense granules (Zarà et al., 2018).

Upon confirming platelet activation, our next objective was to determine if MDA-MB-231 cells internalized mitochondria derived from platelets. It has been reported that activated platelets release mitochondria into the extracellular environment (Boudreau et al., 2014). Using flow cytometry, we demonstrated that MDA-MB-231 cells acquire mitochondria from platelets in an *in vitro* co-culture system. Additionally, confocal microscopy revealed co-localization of platelet-derived mitochondria with endogenous mitochondria in MDA-MB-231 cells. Although we did not establish a connection between platelet-derived mitochondria and the mitochondria of cancer cells, our findings suggest that MDA-MB-231 cells acquire mitochondria from platelets when co-cultured *in vitro*. These results align with recent findings describing the internalization of platelet-derived mitochondria in osteosarcoma (Zhang et al., 2023) and mesenchymal stem cells (MSC) (Levoux et al., 2021).

Our study also utilized MitoTracker™ Deep Red (MTDR), a dye dependent on the mitochondrial membrane potential. In contrast, MitoTracker™ Green (MTG) does not rely on the mitochondrial membrane potential (Agnello et al., 2008; Cottet-Rousselle et al., 2011; Xiao et al., 2016). This dependency raises the possibility that the MTDR dye might not have been effectively retained by throughout the experiment. This could explain why MDA-MB-231 cells exhibit a higher MTG staining than MTDR (Figure 1K). This concern is particularly relevant considering that MDA-MB-231 cells were initially stained with MTDR, co-cultured with MTG-stained platelets, and then analyzed on the third day.

In addition, the more intense staining with MTG could be attributed to the MDA-MB-231 cells exposed to high quantities of platelet-derived mitochondria (Figure 1K). As a result, some of these mitochondria might co-localize with the endogenous mitochondrial network, potentially due to mitochondrial fusion, as observed in the confocal microscopy images (Figure 1K). To verify a connection between the mitochondria of platelets and cancer cells, we could study the morphology of the mitochondrial network in MDA-MB-231 cells cultured individually or with platelets using transmission electron microscopy (TEM). Furthermore, an analysis of protein or gene expression related to mitochondrial dynamics, such as mitofusins, could be analyzed.

Our study shows that incorporating mitochondria from platelets by MDA-MB-231 increases the OCR. However, we did not observe the OCR surpassing the basal rate in all these trials (Figure 2C; Figure 3F). This observation led us to conclude that it is likely that MDA-MB-231 cells consume oxygen at their maximum capacity or that there is a possibility of adjusting the amount of FCCP. We found that 200 nM FCCP is the better concentration for this study and is the concentration used in the seahorse assays (Supplementary Figure S3).

To elucidate the functional impact of acquiring platelet-derived mitochondria by MDA-MB-231 cells, we co-cultured these cancer cells with platelets previously stained with MTG. Using cell sorting, we then separated the cancer cells based on the intensity of the MTG signal, yielding two populations: those with low MTG intensity (MITO-Low) and those with high MTG intensity (MITO-High). As a control, MDA-MB-231 cells cultured alone were subjected to the same cell sorting process. The sorted cancer cells’ relative mitochondrial DNA (mtDNA) content was analyzed by qPCR to authenticate the validity of our experimental approach, which showed differential acquisition of platelet-derived mitochondria by the MDA-MB-231 cells.

On the one hand, we expected that the MITO-Low population had more mitochondria than the PLT-condition. Figure 2I shows that the MITO-High population has greater mtDNA content than the MITO-Low population and PLT-condition. However, no significant differences exist between the MITO-Low and PLT-conditions. This could be explained by the nature of our experimental strategy, in which we used the MTG dye to sort the cells that acquired platelet-derived mitochondria. We thought that there was a possibility that the MITO-Low population acquired cell structures that were MTG positive but were not complete mitochondrion (i.e., structurally damaged mitochondria). If we used platelets with GFP-labeled mitochondria, our experimental strategy could be more accurate, with the expected result of the MITO-Low population with more mitochondria than the PLT-condition. In addition, it has been described that MSC acquires mitochondria from platelets via endocytosis (Levoux et al., 2021). Moreover, cancer cells display notable heterogeneity in gene expression, both *in vitro* and *in vivo,* and alterations in endocytic pathways are frequently seen in cancer cells (Mellman and Yarden, 2013; Kinker et al., 2020). Such variability could lead to a subset of cancer cells possessing enhanced endocytic capabilities, resulting in greater internalization of mitochondria (Patel et al., 2017; Jayashankar and Edinger, 2020).

Our observations underscored that the MITO-High subpopulation of sorted MDA-MB-231 cells had higher ATP content and exhibited more cell proliferation than the MITO-Low subpopulation and the bulk of MDA-MB-231 cells. Conversely, we observed that the ATP content in the MITO-Low subpopulation was lower than in the bulk of MDA-MB-231 cells. Interestingly, the MITO-High subpopulation also had higher mtDNA levels, followed by the PLT-condition and then the MITO-Low subpopulation. The observed effects could be attributed to the amount and quality of platelet-derived mitochondria internalized by the MDA-MB-231 cells. We are exploring the “Mitohormesis” effect, which might yield different outcomes based on various signals like reactive oxygen species (ROS), mitochondrial metabolites, proteotoxic signals, and the mitochondria-cytosol stress response (Kenny et al., 2020). The mitochondria have been recognized as a pivotal organelle within cancer cells (Wallace, 2012). This is further supported by findings that cancer cells devoid of functional mitochondria require more time to form tumors than those with functional mitochondria (Tan et al., 2015). In addition, through the tricarboxylic acid cycle (TCA), mitochondria generate molecules that serve as the building blocks for synthesizing essential macromolecules, aiding in the proliferation of cancer cells (Vasan et al., 2020). Consequently, it is conceivable that the MITO-High subpopulation possesses a superior capacity for metabolite and ATP production, which might facilitate a more active state of cell proliferation. This supposition aligns with recent studies, where it was noted that glioblastoma cells with newly acquired exogenous mitochondria displayed increased cell proliferation (Watson et al., 2023).

On the other hand, elevated platelet counts during diagnosis often signal poorer outcomes, particularly in breast cancer (Giannakeas et al., 2021; Tao et al., 2021). This leads us to hypothesize that platelets play a pivotal role in the pathophysiology of hematogenous metastasis. This hypothesis is corroborated by the widespread expression of the clot-initiating protein, tissue factor (TF), across various solid tumors, a factor typically associated with advanced stages of the disease (Gomes et al., 2017). Given these insights, it is plausible that therapeutic strategies aimed at reducing platelet count or inhibiting their activation could slow cancer progression and improve patient outcomes. This perspective opens avenues for exploring pharmacological strategies that target platelets, which may significantly influence the course of cancer. Within this context, drugs that modulate platelet activation could be potential tools for mitigating TNBC progression.

## Data Availability

The original contributions presented in the study are included in the article/Supplementary Materials, further inquiries can be directed to the corresponding authors.

## Supplementary material

SUPPLEMENTARY FIGURE S1 Gating strategies for analyzing flow cytometry data. **(A)** Flow cytometry analysis was used to evaluate the internalization of mitochondria by MDA-MB-231 cells. MDA-MB-231 cells were selected using the SSC-A v/s FSC-A plot. Double events were excluded via the FSC-H v/s FSC-A plot. MDA-MB-231 cells positive for CellTrace^™^ Violet (CTV) were gated using the FSC-A v/s CTV plot. The internalization of mitochondria was analyzed using flow cytometry histograms and median fluorescence intensity (MFI) for MitoTracker^™^ Green (MTG). **(B)** Flow cytometry analysis evaluated mitochondrial membrane potential and morphological changes in MDA-MB-231 cells. MDA-MB-231 cells were selected using the SSC-A v/s FSC-A plot. Double events were discarded via the FSC-H v/s FSC-A plot. MDA-MB-231 cells positive for CTV were gated using the FSC-A v/s CTV. Relative cell size and complexity were analyzed using flow cytometry histograms MFI for FSC-H and SSC-H, respectively. Then, live MDA-MB-231 cells were identified using the FSC-A compared to the live/dead plot from flow cytometry histograms. The mitochondrial membrane potential was analyzed using median fluorescence intensity (MFI) for TMRE. **(C)** Flow cytometry analysis was utilized to assess platelet activation. Platelets were selected using the SSC-A v/s FSC-A plot. Double events were not discarded due to the impact of platelet activation on their morphological properties. Platelets positive for CD42a were selected using the FSC-A v/s CD42a plot. Platelet activation was analyzed by measuring CD62p expression using the FSC-A v/s CD62p plot, flow cytometry histograms, and MFI for CD62p.

SUPPLEMENTARY FIGURE S2 Evaluation of MitoTracker™ Green leakage from platelets to MDA-MB-231 cells. Transwell™ (TW) experiments (TW+) were performed to evaluate the potential leakage of MitoTracker™ Green (MTG) from platelets (PLT) to MDA-MB-231 cells. MTG-stained platelets were placed in the upper compartment, and MDA-MB-231 cells were placed in the lower compartment of the Transwell™ inserts (TW+) (pore size of 0.4 µm) for 24 h. In addition, we used PLT stained with MTG in co-culture with MDA-MB-231 cells (TW-) as a control for 24 h. The potential leakage of MTG from PLT to MDA-MB-231 cells was analyzed using flow cytometer. The histograms and median fluorescence intensity (MFI) of MTG signal in MDA-MB-231 cells are shown. A t-student statistical test was performed **p* < 0.05.

SUPPLEMENTARY FIGURE S3 Determination of optimal FCCP concentration for the Agilent Seahorse XF Cell Mito Stress Test. Seahorse experiments were performed to determine the optimal concentration of FCCP for seahorse trials. MDA-MB-231 cells were seeded on XF HS Mini 8-well plates with DMEM-HG. After 48 h, the cell culture medium was replaced with Agilent Seahorse XF DMEM medium pH 7.4 containing 10 mM glucose. Mitochondrial function was evaluated using 2 µM oligomycin (Oligo), a range of FCCP concentrations [100 nM (FCCP 1), 150 nM (FCCP 2), 200 nM (FCCP 3), 300 nM (FCCP 4), and 400 nM (FCCP 5)], 1 µM rotenone (Rot), and 1 µM antimycin-A (AA).
