# Supplementary figures and images for: Impact of platelet-derived mitochondria transfer in the metabolic profiling and progression of metastatic MDA-MB-231 human triple-negative breast cancer cells
Source: Front Cell Dev Biol. 2024 Jan 12;11:1324158. doi: 10.3389/fcell.2023.1324158 (PMC10811077; doi:10.3389/fcell.2023.1324158)

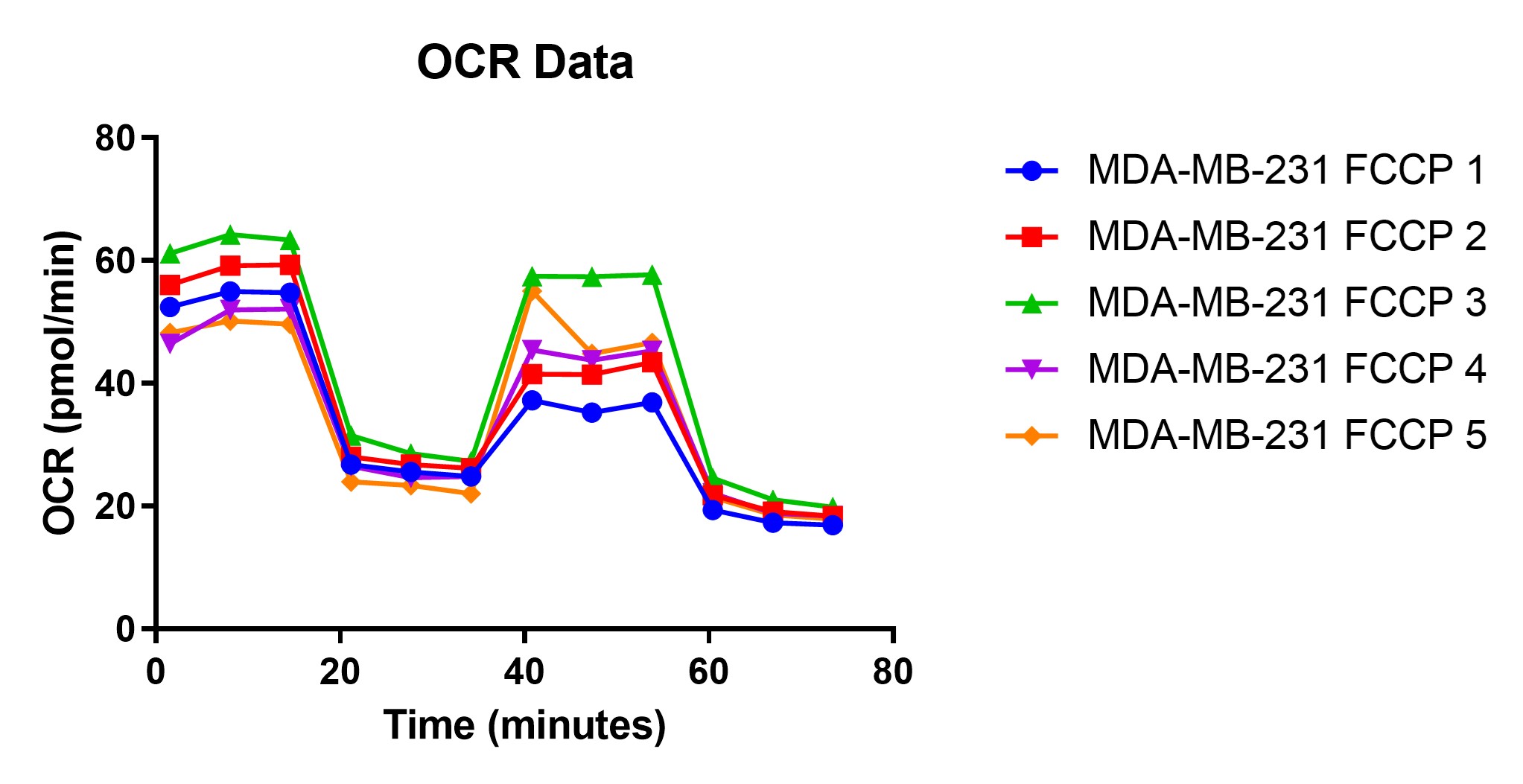

Supplement: Supplementary file 1 [file Image3.jpeg]

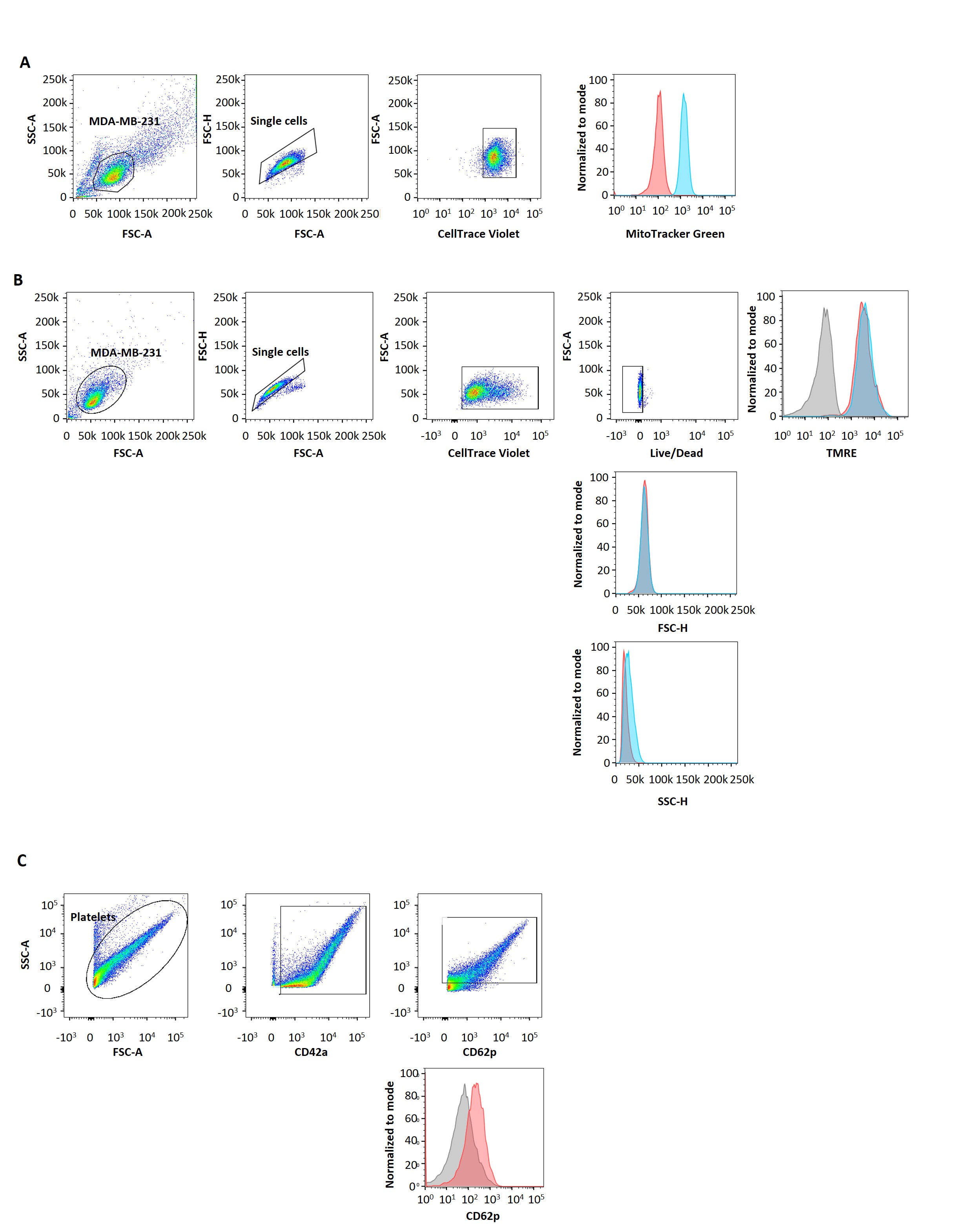

Supplement: Supplementary file 2 [file Image1.jpeg]

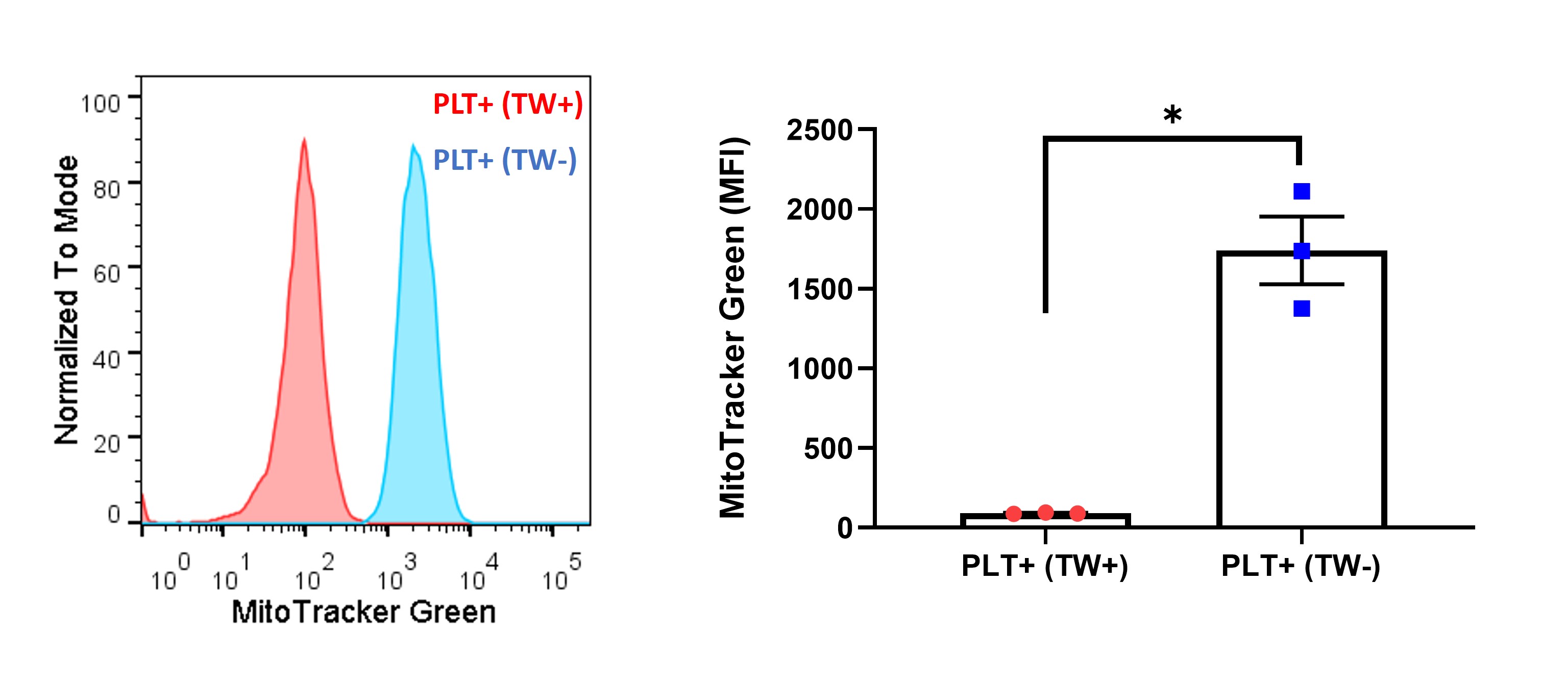

Supplement: Supplementary file 3 [file Image2.jpeg]
